# The association between different domains of quality of life and symptoms in primary care patients with emotional disorders

**DOI:** 10.1038/s41598-018-28995-6

**Published:** 2018-07-25

**Authors:** César González-Blanch, Fernando Hernández-de-Hita, Roger Muñoz-Navarro, Paloma Ruíz-Rodríguez, Leonardo Adrián Medrano, Antonio Cano-Vindel

**Affiliations:** 1 Mental Health Centre, University Hospital “Marqués de Valdecilla”- IDIVAL, Santander, Spain; 2 Faculty of Health Sciences, Universidad Europea del Atlántico, Santander, Spain; 3 Department of Basic Psychology, Faculty of Psychology, University of Valencia, Valencia, Spain; 4 Castilla La Nueva Primary Care Centre, Health Service of Madrid, Madrid, Spain; 5 Faculty of Psychology, University Siglo 21, Córdoba, Argentina; 6 Department of Basic Psychology, Faculty of Psychology, University Complutense of Madrid, Madrid, Spain

## Abstract

Despite the importance of quality of life (QoL) in primary care patients with emotional disorders, the specific influence of the symptoms of these disorders and the sociodemographic characteristics of patients on the various QoL domains has received scant attention. The aim of the present study of primary care patients with emotional disorders was to analyse the associations between four different QoL domains and the most prevalent clinical symptoms (i.e., depression, anxiety and somatization), while controlling for sociodemographic variables. A total of 1241 participants from 28 primary care centres in Spain were assessed with the following instruments: the Patient Health Questionnaire (PHQ)-9 to evaluate depression; the Generalized Anxiety Disorder Scale (GAD)-7 for anxiety; PHQ-15 for somatization; and the World Health Organization Quality of Life Instrument-Short Form (WHOQOL-Bref) to assess four broad QoL domains: physical health, psychological health, social relationships, and environment. The associations between the symptoms and QoL domains were examined using hierarchical regression analyses. Adjusted QoL mean values as a function of the number of overlapping diagnoses were calculated. The contribution of sociodemographic variables to most QoL domains was modest, explaining anywhere from 2% to 11% of the variance. However, adding the clinical variables increased the variance explained by 12% to 40% depending on the specific QoL domain. Depression was the strongest predictor for all domains. The number of overlapping diagnoses adversely affected all QoL domains, with each additional diagnosis reducing the main QoL subscales by 5 to 10 points. In primary care patients with a diagnostic impression of an emotional disorders as identified by their treating GP, clinical symptoms explained more of the variance in QoL than sociodemographic factors such as age, sex, level of education, marital status, work status, and income. Given the strong relationship between depressive symptoms and QoL, treatment of depression may constitute a key therapeutic target to improve QoL in people with emotional disorders in primary care.

## Introduction

The WHO defines quality of life (QoL) as an individual’s perception of their position in life embedded in a cultural, social, and environmental context^1^. The concept of QoL is broad and is composed of numerous domains, including the physical, psychological, social, family and environmental domains. Evaluation of these domains can reveal the overall impact of illness on a patient’s life. Importantly, QoL has been shown to contribute more to an individual’s perception of their wellbeing, health, and life satisfaction than objective measures of life conditions^2^.

In the last three decades, there has been an exponential rise in the number of studies performed to investigate QoL in nearly all areas of medicine^3^. This growing interest has been especially notable in the fields of psychiatry and psychology to assess the role of QoL in mental disorders. Studies have been conducted to assess the impact of QoL in schizophrenia^4,5^, depression^6^ and anxiety disorders^7^, for general health^8^ and to determine the role of QoL for mental health services recommendations^9^. In parallel, mental health services have shifted away from focusing on symptom reduction towards a more holistic approach encompassing other factors such as wellbeing and functioning. This shift has occurred largely due to the publication of studies on the impact of mental disorders in QoL. For example, QoL has shown to be a better predictor of sustained remission than symptom resolution in depression, leading some authors to suggest that QoL should be the ultimate goal of treatment in these patients^10^.

Studies have shown that QoL is associated with the psychopathology severity^11^, and QoL is known to worsen as a function of the number of comorbid mental disorders^12,13^. Common mental disorders in primary care such as mood, anxiety, and somatoform disorders are associated with a greater decline in QoL than medical disorders such as diabetes or heart disease^14–16^.

Previous studies have described the relationship between various QoL domains and a range of sociodemographic factors, including age, gender, occupation, income, marital status, and educational level^17–22^. However, most of those studies were conducted in elderly populations or in patients with severe medical conditions such as cancer, rheumatological diseases, or kidney conditions. Moreover, those studies have reported conflicting results, probably due to the heterogeneity of the populations assessed. Although QoL correlates closely with severe mental illness, particularly schizophrenia^23–26^, in people with common mental disorders, the impact of symptoms or sociodemographic characteristics on QoL is not well-understood.

In order to promote the health and wellbeing of patients with common mental disorders, it is crucial to understand how the various sociodemographic factors and specific symptoms effect the various QoL domains. For example, it would be highly beneficial to known whether specific clinical symptoms have a unique contribution— beyond the influence of sociodemographic variables—to specific QoL domains, as such information could have far-reaching clinical implications with regard to assessment and treatment of common mental disorders in primary care. Indeed, this would provide a compelling reason to ensure that primary care services should be modified so as to greatly augment the provision of the effective treatments, according to clinical guidelines, as soon as possible.

Emotional disorders —which in this study include depressive, anxiety, and somatoform disorders— are highly prevalent in primary care patients. Although the prevalence of emotional disorders can vary substantially between studies even when the same diagnostic instrument is used, one of the largest epidemiological studies of mental disorders in primary care performed in a European country found that the three most prevalent emotional disorders in Spain were mood (35.8%), anxiety (25.6%), and somatoform (28.8%) disorders^27^. In general population studies, prevalence rates for these disorders are lower, but still substantial^28^. Given that approximately one-third of primary care visits are due to explicit psychological problems such as full-blown depressive syndrome, anxiety, or somatoform disorder^29^, and that up to 90% of people diagnosed with common mental disorders are treated by a general practitioner (GP)^30^, it is clear that this is the ideal population to carry out such research. Moreover, PC consultations in most European countries last only a mean of 10 minutes, and even less in some countries (e.g., Spain and Germany) where, due to high caseloads, consultation times can be as low as 7–8 minutes^31^.

In this context, the present study had two primary aims. The first aim was to determine the levels of QoL associated with emotional disorders in primary care patients. The second aim was to analyse the associations between several different QoL domains and the most prevalent clinical symptoms (i.e., depression, anxiety, and somatization) after controlling for sociodemographic variables. We hypothesized that (1) QoL —even in primary care patients exhibiting clinically-significant symptoms of emotional disorders but without fulfilling diagnostic criteria— would be substantially impaired, and would worsen significantly as a function of the number of diagnoses they met, and (2) that QoL would show domain-specific associations with clinical symptoms.

## Materials and Methods

### Participants

We analysed data from 1241 individuals participating in the ongoing Psychology in Primary Care (PsicAP) study conducted at 28 primary care centres located in eight regions in Spain (Madrid, Valencia, Basque Country, Castilla La Mancha, Balearic Islands, Cantabria, Navarre, and Galicia). The PsicAP was designed to test the efficacy of psychological treatments for emotional disorders in primary care through a randomized controlled trial see^32^. We included all consecutive eligible 18- to 65-year-old patients who, in the opinion of the treating GP, were considered to have some type of emotional distress (i.e., anxiety, depression or somatization). All individuals who agreed to participate in the PsicAP study were randomized between January 2014 and May 2017. The GPs were advised to exclude patients who they believed should be referred to specialized care (i.e., those with severe mental disorders such as psychosis, eating or personality disorders, substance abuse, or dependence disorders). Patients with insufficient Spanish language skills or an intellectual disability were also excluded. The study procedure was as follows. First, the treating GP reached an an initial diagnostic impression based on th e usual procedures (mainly clinical interview). Next, the participating GPs invited patients to participate in a clinical trial at the centre, and provided them with an Information Sheet containing details about the study, together with an informed consent form for them to sign if they wished to participate. All participants that agreed to participate and signed the informed consent form were then scheduled to meet with a clinical psychologist within the next three weeks. Then, participants completed all self-report measures using computerized versions of these instruments. In this study, only pretreatment data were used before patients were randomized. The ethics committees at each centre, the National Ethics Committee, and the Spanish Agency of Medicines and Medical Devices (AEMPS) all approved the study protocol (code: ISRCTN58437086). All participants provided written informed consent. All procedures were performed in accordance with relevant guidelines and regulations.

### Measures

#### Depression

Depression was assessed with the nine-item Patient Health Questionnaire (PHQ) depression module (PHQ-9)^33,34^. The PHQ-9 is a specific screening test for depression. Each item corresponds to the nine DSM-IV criteria for the diagnosis of major depressive disorder (MDD), which considers symptoms experienced during the two weeks prior to the test administration. Each item is scored on a Likert scale from 0 to 3 (0 = not at all; 1 = several days; 2 = more than half of the days; 3 = almost every day), thus the total scores can range from 0 to 27 points. For a diagnosis of MDD, the patient must score 2 or 3 points on at least one of the two first symptoms and 2 or 3 points on at least 5 of the 9 items. The diagnostic algorithm of the Spanish version of the PHQ-9 has acceptable sensitivity (0.88) and specificity (0.80) in primary care centres in Spain^35^. In this study, the PHQ-9 had a Cronbach’s alpha of 0.87, indicating good internal consistency.

#### Anxiety

Anxiety was assessed with the seven-item PHQ Generalized Anxiety Disorder Scale (GAD-7)^36,37^. The GAD-7 consists of 7 items that assess the presence of anxiety symptoms in the last two weeks through a four-point (scored from 0 to 3) Likert scale according to the frequency of symptoms in that time period (0 = not at all: 1 = several days; 2 = more than half of the days; 3 = almost every day), with a maximum score of 21 points. The most widely used cut-off score for a diagnosis of GAD is 10, with a sensitivity of 0.87 and a specificity of 0.78 in a Spanish primary care population^38^. In this study, the GAD-7 had a Cronbach’s alpha of 0.87, indicating good internal consistency.

#### Somatization

Somatic symptom severity was assessed with the 15-item PHQ somatic symptom severity scale (PHQ-15)^39,40^. The PHQ-15 is a screening test for somatization disorder and includes 14 of the 15 most prevalent DSM-IV somatic symptoms of somatization disorder. In the Spanish version, patients are asked to rate the severity of 13 somatic symptoms over the last four weeks, scored from 0 to 2 as follows: 0 (not bothered), 1 (bothered a little), or 2 (bothered a lot). Two additional somatic symptoms from the PHQ-9 (fatigue and sleep complaints) are scored according to frequency as follows: 0 points (“not at all”), 1 (“several days”), or 2 (“more than half the days” or “nearly every day”). The total PHQ-15 score ranges from 0 to 30. A diagnosis of somatoform disorder requires a score of 2 (without a biological explanation) on at least three of the first 13 symptoms based on a cut-off point of 10. Based on these criteria, the PHQ-15 has a sensitivity of 0.78 and specificity of 0.71^41^. The PHQ-15 had a Cronbach’s alpha of 0.80 in the present study, indicating good internal consistency.

#### Quality of Life (QoL)

The World Health Organization Quality of Life Instrument-Short Form (WHOQOL-Bref) was used to assess QoL^1^. The WHOQOL-Bref is an abbreviated version of the WHOQOL-100 (WHO, 1998). This brief version of the scale consists of 26 questions, with response options on a five-point scale. The first two questions are general, and the remaining 24 represent each of the four main domains (physical, psychological, social relations and environment). Each sub-scale is scored positively and then normalised for comparability with the WHOQoL-100. We also used the first question of the WHOQOL-Bref, which assesses overall QoL on scale of 0 to 4. Higher scores correspond to a higher QoL. The validation study of the WHOQOL-Bref in Spain, including data from primary care centres, yielded positive evidence for acceptability, internal consistency, and convergent and discriminant validity^42^.

### Data analyses

Descriptive statistics were calculated for the sample’s socio-demographic, clinical, and QoL profiles. Categorical variables are presented as percentages and continuous variables as means (± standard deviation [SD]) or medians (interquartile range [IQR]). Assumptions of normal distribution were explored with the Kolmogorov–Smirnov test and visual inspections of the histograms. We used the Pearson or Spearman correlation coefficients, as appropriate, to examine the relationships between variables.

A series of hierarchical multiple linear regression analyses were conducted to determine the impact of clinical symptoms (i.e., anxiety, depression and somatizations) on QoL. In these models, sociodemographic data (age, sex, education, marital status, employment status, and income) were entered in Step 1 and clinical symptoms were entered jointly in Step 2. Dummy variables were created for the independent variables with a nominal or ordinal level of measurement. To address the skewed data distributions, we carried out a bootstrapping analysis in our regression models by calculating bias-corrected and accelerated (BCa) confidence intervals (CI) with 1,000 replications. Bootstrapping is a nonparametric procedure that provides reliable measures of statistical significance, even when data do not follow a standard parametric distribution^43^. The values of the samples are used to estimate the 95% confidence interval (CI) for the parameter. An effect is considered significant when the BCa CI does not include a zero. Tolerance and variance inflation factor (VIF) were used to check for multicollinearity. Tolerance values of less than 0.10 and VIF values greater than 10 typically indicate multicollinearity issues^44^.

MANCOVA was used to examine differences between QoL domains in four categories as a function of the number of overlapping diagnoses (0, 1, 2, and 3 mental disorder diagnoses: MDD, GAD, and/or somatoform disorder). We used the diagnostic algorithms and cut-off scores of the PHQ-9, GAD-7, and PHQ-15, respectively, to establish the diagnosis of MDD, GAD and somatoform disorders. Sociodemographic variables were entered as covariates, and adjusted means scores were reported. All statistical analyses were two-tailed, and, because multiple comparisons were performed, the alpha level was set at 0.01. All analyses were carried out using SPSS 20 for Windows (SPSS Inc., Chicago, Illinois).

### Data Availability

The datasets analyzed during the current study are available from the corresponding author on reasonable request.

## Results

### Sample description

**Figure 1:**
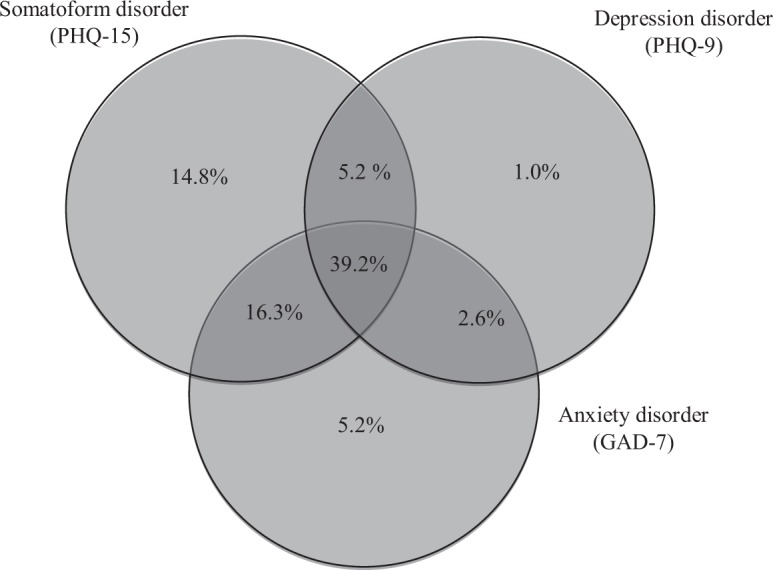
The overlap of major depression, generalized anxiety and somatoform disorders (based on algorithms from the PHQ-9, GAD-7 and PHQ-15, respectively). Values indicate the percentage of the total sample (n = 1241).

The mean age of the 1241 primary care patients was 43.2 years (SD, 12.1). Most patients were female (77%) and married (61.2%). Most (48%) had a middle or high school educational level. Just over half (51%) were employed and 42% earned less than 12,000 €/year (see Table 1). The mean values for the PHQ-9, GAD-7 and PHQ-15 ranged from 10–14 points, indicating moderate symptom intensity based on the cut-off points^45^. The diagnostic overlap of these three disorders, based on their respective scale algorithms, is shown in Fig. 1. The mean and median values for each domain on the WHOQOL-Bref scale are shown in Table 2. Half of the patients scored ≤50 on the main domains of the WHOQOL-Bref.

**Table 1:** Sociodemographic and clinical characteristics of the participants (n = 1241).

	N	%
*Sex*		
Female	956	77
Male	285	23
*Age* (mean ± SD)	43.2	12.1
*Age group*		
18–25	118	9.5
26–35	241	19.4
36–50	482	38.8
51–65	400	32.2
*Marital status*		
Single	255	20.5
Married	760	61.2
Divorced	192	15.5
Widowed	34	2.7
*Education*		
Primary	351	28.3
Secondary	589	47.5
University	301	24.3
*Employment status*		
Employed	634	51.1
Unemployed	440	35.5
Medical leave	102	8.2
Retired	65	5.2
*Income*		
<12000€	524	42.2
12000–24000€	491	39.6
24000–36000€	157	12.7
>36000€	69	5.6
*Clinical variables*^a^ (mean ± SD)		
Depression (scale 0 to 27)	13.6	6.5
Anxiety (scale 0 to 21)	11.7	5.2
Somatization (scale 0 to 30)	14.2	6.0

^a^Depression, anxiety and somatization intensity as measured by the PHQ-9, GAD-7 and PHQ-15, respectively.

**Table 2:** QoL (as measured with the WHOQOL-Bref).

	Mean	SD	Median	Range
***QoL***				
Overall	2.9	0.9	3	2–3
Psychological domain	54.2	17.2	56	44–63
Physical health	44.8	17.4	44	31–56
Social relationships	50.0	20.7	50	31–69
Environment	56.1	14.8	56	44–69

WHOQOL-Bref, World Health Organization Quality of Life. Instrument-Short Form; SD, standard deviation.

### Association between sociodemographic variables and QoL

The contribution of sociodemographic variables to most QoL domains was modest, explaining anywhere from 2% to 11% of the variance, depending on the specific variable. Gender was a significant variable only in the psychological domain model. Age contributed significantly to both the psychological and social domains. Education was a significant variable only for the environmental domain. Employment was significant on both the psychological and physical domains. Annual income contributed to the environmental domain, and was the only sociodemographic variable included in the overall QoL score.

### Association between clinical symptoms and QoL

On the univariate analysis, depression (measured by PHQ-9), anxiety (GAD-7), and somatizations (PHQ-15) were all highly correlated with QoL domains. The magnitude of the effect size (r values) for the relationship between depression intensity and QoL domains ranged from 0.38 to 0.67, depending on the specific domain. The effect sizes for the association between anxiety intensity and QoL domains ranged from 0.24 to 0.51. The correlation between somatization intensity and QoL dimensions ranged from 0.20 to 0.56 (see Table S1).

To determine the extent to which clinical symptoms were independently related to the various QoL domains, we conducted a series of separate hierarchical multiple linear regression analyses for each outcome variable. Sociodemographic variables (i.e., age, sex, education, employment status, and income) were entered in the first step, and the clinical variables (i.e., PHQ-9 depression, GAD-7 anxiety, and PHQ-15 somatizations) were entered in the second step. As indicated above, multicollinearity was assessed by examining the tolerance and VIF statistics for each variable within a regression model. The Tolerance values ranged from 0.38 to 0.91 and the VIF values ranged from 1.10 to 2.66, thus suggesting that multicollinearity had no significant impact on any of the variables in the analyses. Table 3 shows the results of the regression models on the overall QoL score and for each QoL domain. The sociodemographic variables had only a weak influence in the models, whereas the addition of clinical variables significantly increased the variance explained in each model, particularly on the psychological and physical domains of QoL (Δ R^2^ values of 0.35 and 0.40, respectively), in which all clinical symptoms contributed independently. Depression intensity was the best predictor on all QoL domains, indicating, as expected, that more severe depression was associated with poorer QoL.

**Table 3:** Hierarchical multiple linear regression analysis examining the contribution of sociodemographic and clinical variables to each QoL domain (as measured with the WHOQOL-Bref)^a^.

	Overall QoL	Psychological	Physical	Social relations	Environmental
Predictors	**β**	**β**	**β**	**β**	**β**
**Sex**	−0.02	−0.07**	0.05	−0.03	0.01
**Age**	−0.05	−0.10**	0.03	−0.11**	0.06
**Marital status** (ref: single)					
married	0.02	0.01	0.07	0.10	0.03
divorced	0.03	−0.01	0.02	0.04	−0.05
widowed	0.03	0.03	0.01	0.04	0.07
**Education** (ref: lowest)					
middle	0.03	0.00	0.05	−0.05	0.06
high	0.02	−0.04	0.07	−0.06	0.11*
**Employment status** (ref: employed)					
unemployed	−0.07	−0.14**	−0.08**	−0.01	−0.02
on sick leave	−0.06	−0.16**	−0.03	0.04	−0.01
retired	0.01	−0.08*	−0.01	0.03	0.00
**Income**^b^ (ref: <12€):					
12–24€	0.03	0.01	−0.01	0.03	0.09**
24–36€	0.04	0.06	0.04	0.04	0.11**
>36€	0.09*	0.08*	0.05	0.03	0.15**
**Clinical symptoms**					
Depression (PHQ-9)	−0.40**	−0.30**	−0.64**	−0.49**	−0.23**
Anxiety (GAD-7)	−0.04	−0.11**	−0.11**	0.03	−0.14**
Somatization (PHQ-15)	0.04	−0.29**	0.10*	0.10	−0.03
Step 1 (R^2^)	0.046	0.114	0.083	0.023	0.107
Step 2 (Δ R^2^)	0.153	0.350	0.400	0.160	0.118
Model significance (Step 2)	F_16,1219_ = 18.88**	F_16,1219_ = 66.03**	F_16,1219_ = 79.93**	F_16,1219_ = 17.07**	F_16,1219_ = 22.13**

*p < 0.01; **p < 0.001.

WHOQOL-Bref, World Health Organization Quality of Life Instrument-Short Form;

^a^Predictors data corresponding to Step 2.

^b^In thousands of Euros.

### Impact of the number of mental disorders on QoL

**Figure 2:**
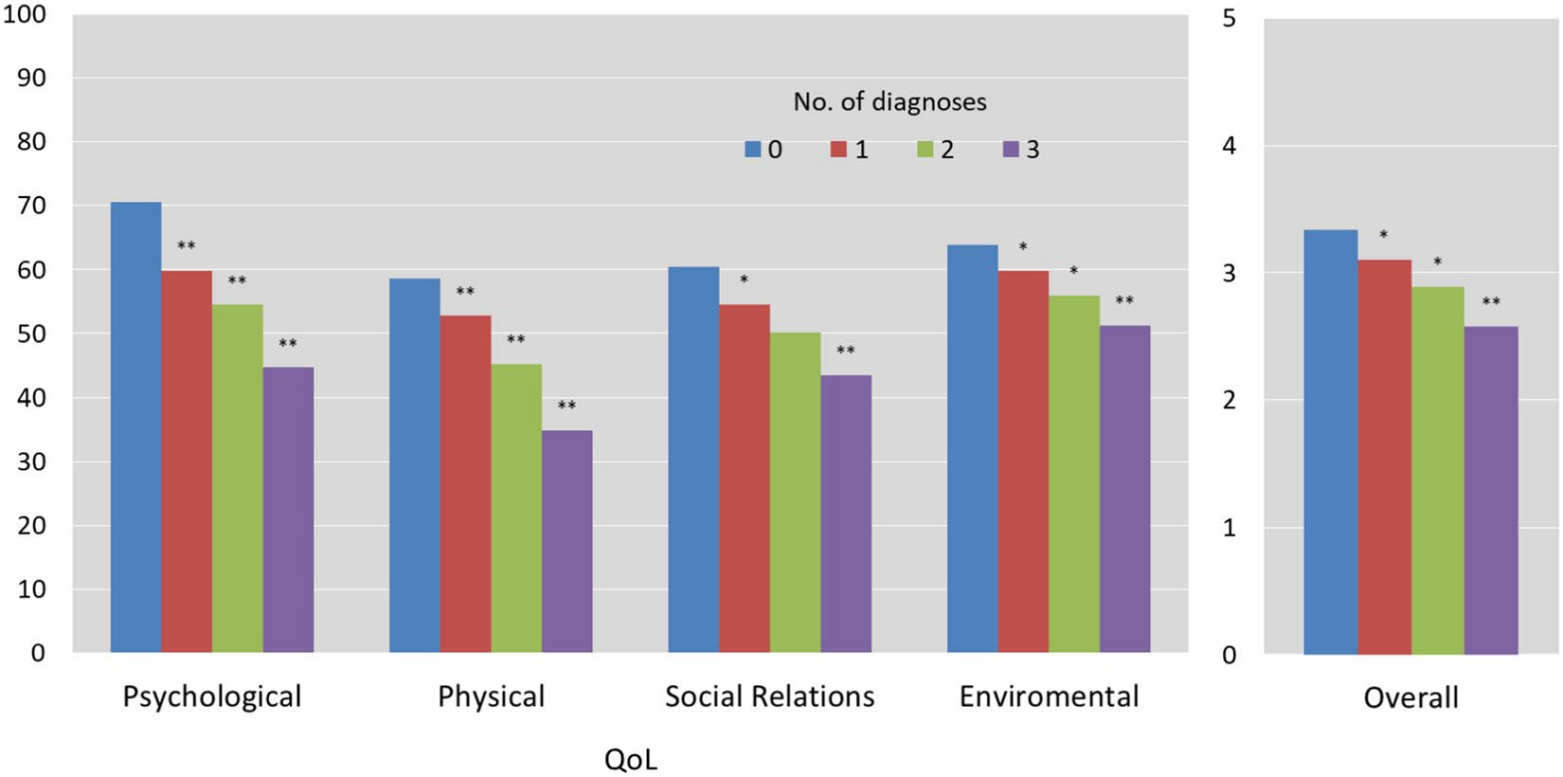
Covariate Adjusted Means of QoL as a function of the number of mental disorder diagnoses^b^. *p < 0.01; **p < 0.001. WHOQOL-Bref, World Health Organization Quality of Life Instrument-Short Form; QoL, Quality of Life. ^a^Means after controlling for covariates (age, sex, education, marital status, employment status, and income). ^b^Significance in the column ‘1 diagnosis’ refer to the difference with ‘no diagnosis’, in the ‘2 diagnoses’ column, to the difference between 2 diagnoses and 1 diagnosis, and in the ‘3 diagnoses’ column to the difference between 3 diagnoses and 2 diagnoses. Diagnoses based on algorithms from the PHQ-9, GAD-7 and PHQ-15 for major depression, generalized anxiety and somatoform disorders, respectively. ^b^SE- standard errors based on 1000 bootstrap samples. ^c^In parenthesis overall score range of each subscale.

We also tested whether there were differences in the QoL domains as a function of the number of mental disorders. Pillai’s trace statistic showed a significant effect for the number of diagnoses on the QoL domains, V = 0.37, F(15, 3675) = 34.84, p < 0.001, multivariate ηp² = 0.13, which can be interpreted as a medium to large effect. Figure 2 illustrates the negative, proportional effect of the number of mental disorder diagnoses on QoL domains: on average, each additional diagnosis reduced the score on each of the main QoL subscales by 5 to 10 points (see Table S2).

## Discussion

The present study was conducted in a large sample (n = 1241) of primary care patients with a diagnostic impression of depression, anxiety, or somatization not requiring referral to specialized care. The intensity of these mental disorders was significantly associated with QoL domains. More specifically, the symptoms of all three disorders were independently associated with the psychological and physical domains of QoL while only depression intensity was significantly associated with the social relations domain. Moreover, clinical symptoms explained a far greater percentage of variance than any of the sociodemographic variables (age, sex, education, marital status, work status, and income). Of all the sociodemographic variables and clinical symptoms assessed, depression was the best predictor of QoL. Diagnostic overlap, which was common in this sample, contributed to a further worsening on all QoL facets assessed in the study.

Our data show that even the patients in our sample who did not meet the diagnostic criteria for a mental disorder, had worse QoL scores than normal populations (as reported in the WHOQOL-Bref in preliminary population norms^46^ and compared to data from a meta-analysis adjusted to the country’s Human Development Index^47^. Moreover, in patients with at least one mental disorder, QoL was more than one standard deviation lower than the general population, and QoL decreased by 5–10 points (WHOQoL-Bref scale) for each additional disorder.

Overall, these results are consistent with findings from previous studies that have investigated the association between common mental disorders and QoL in primary care^14,15,48^ and general population samples^49,50^. In this sense, our study extends these findings to a sample of primary care patients with a suspected diagnosis of an emotional disorder not sufficiently severe to require specialized care. This is a particularly relevant population given that such patients are highly prevalent in primary care settings, in which treatment is often delivered by GPs who have a high caseload^31^; as a consequence of this caseload, treatment is often inadequate, as several studies have shown^51,52^.

Findings from studies comparing the relative impact of each symptom on functioning and QoL are mixed. While most studies have found that depression has a greater impairment^49,50,53–57^, other studies have reported that the pattern of impairment depends on the specific symptom^48,58^, and other studies have found that the effects of anxiety disorders and depression are similar^59,60^. On the univariate analyses, depression, anxiety, and somatization correlated with all QoL domains. By contrast, the regression models showed significant associations between anxiety and QoL on only three domains (psychological, physical and environmental). Somatizations contributed only to the psychological and physical domains of QoL. The large effect of depression on all QoL domains might arguably be due to what some authors have called ‘tautological measures’^61^, suggesting a substantial overlap between the content of scales used to measures both depression and QoL. By contrast, Trompenaars *et al*.^62^ found that the common variance between depression and QoL did not exceed 25%. Likewise, none of the clinical symptoms explained more than 40% of the variance on any of the regression models in our study. The robustness of the association between depression and QoL is supported by the significant impact of depression on all QoL facets, including those related to social relations and environmental domains. However, it must be noted that for social relations and environmental aspects of QoL, clinical symptoms contributed only 16% and 12%, respectively, to the model variance, which is considerably less than the contribution of clinical symptoms on the psychological and physical domains (35% and 40%, respectively). This finding suggests that alleviation of clinical symptoms may not be the only solution to improving the many aspects of well-being.

On the regression models performed in our study, the sociodemographic variables—age, sex, educational level, marital status, work status, and income—had distinctive pattern of associations with QoL. Given the methodological differences between the studies conducted to date to evaluate the influence of these variables on QoL, it is difficult to compare our results to previous reports. However, other studies carried out in the primary care setting that have analysed demographic variables have also found differences in the association between such variables and QoL domains. For example, Brenes (2007) used the Medical Outcomes Study Short-Form 36-Item Health Survey (SF-36), finding that age, race, education, marital status and gender were differently associated with each one of the 8 domains assessed by the SF-36. Similarly, García-Campayo *et al*. (2008), using an abbreviated version of the SF-36, found that age was significantly associated with the mental component of QoL, but not with the physical domain. Importantly, these sociodemographic variables contributed substantially less to the variance of QoL domains than clinical variables; the only exception was for the environmental domain, in which sociodemographic and clinical variables accounted for a similar percentage of variance (11 vs 12%).

The findings presented here may have important clinical and research implications in primary care, particularly given the tendency of GPs to focus on symptom relief while largely overlooking disability and QoL. This is important because patients with emotional disorders—even those in whom specialized care may not be indicated— may have marked impairment on multiple QoL domains that are only partially explained by symptoms. If restoration of normal functioning and wellbeing are considered the ultimate therapeutic goal, the results of the current study further support the use of more comprehensive outcome measures in both clinical practice and in research protocols. At present, it is unclear whether the psychological and pharmacological treatments in routine practice—which are primary aimed at symptom alleviation—can improve other important outcomes such as QoL. Moreover, given the high rates of comorbidity for anxiety, depression, and somatization in this population, transdiagnostic approaches may be a particularly effective method of treating emotional disorders. Indeed, a recent meta-analysis of transdiagnostic psychological treatments for anxiety and depressive disorders found that such an approach yielded notable improvements (medium-sized effects) in QoL outcomes^63^. Likewise, a more recent meta-analysis performed to evaluate the effect of treatments for depression on QoL found that cognitive-behavioral therapy (CBT) achieved moderate improvements in QoL that remained stable over time. It is worth emphasizing that the improvement in depression symptoms was positively associated with changes in QoL only for patients who received CBT but not for those who received pharmacological treatment^64^. A plausible explanation for the notable but modest improvement in QoL compared to the decrease in symptoms might be that these interventions only help with some aspects of patients’ wellbeing (i.e., psychological and physical domains), but might be less than satisfactory for others (i.e., social relations and environmental domains).

This study has several limitations. First, it was a cross-sectional study and thus we cannot make any inferences about causality or directionality; several authors have found a bidirectional relationship, with anxiety and depression symptom severity predicting functional impairment while functional impairment predicts anxiety and depression symptom severity^65^. Second, we cannot rule out selection bias, as the study included only a subgroup of patients (that is, those willing to participate in a clinical trial). As a result, the participants may not be representative of all primary care patients with emotional disorders but rather they may represent only a subgroup of patients who are particularly concerned about their mental health. Thus, in a sample without any selection bias, QoL may be lower. Furthermore, although this is a large sample obtained from multiple centers from several different regions in Spain, the use of convenience sampling might limit the generalizability of the results to all primary care patients with emotional disorders. Third, symptoms were assessed with self-report measures; while such measures are considered both valid and reliable—and widely-used for mental health screening in primary care settings—these types of instruments may have reporting biases and, therefore, should not be interpreted as clinical diagnoses but rather as indicative of potentially clinically-significant symptoms. Finally, another limitation of our study was the lack of data on physical comorbidities or other treatments.

### Conclusion

The findings of the present study involving primary care patients with emotional disorders detected by the treating GP indicate that symptom intensity and comorbidity are associated with a marked decrease in various QoL domains. Depression had a particularly strong association with QoL, and this association remained significant on all QoL domains, even after adjusting for potential sociodemographic confounders, which, in turn, showed a distinctive and attenuated pattern of associations with QoL. Given the modest variance explained by clinical symptoms on the social and environmental domains, in seems clear that while treating clinical symptoms is necessary, this might be insufficient to achieve full recovery as measured by improved QoL outcomes in patients with common mental disorders.

## Electronic supplementary material

Supplementary Material
