# Supplementary material for: The association between different domains of quality of life and symptoms in primary care patients with emotional disorders
Source: Sci Rep. 2018 Jul 25;8:11180. doi: 10.1038/s41598-018-28995-6 (PMC6060102; doi:10.1038/s41598-018-28995-6)
Supplement: Supplementary file 1 — Supplementary Material [file 41598_2018_28995_MOESM1_ESM.pdf]

The association between different domains of quality of life and symptoms in primary care patients with emotional disorders

César González-Blanch[1,2]

Fernando Hernández de Hita[1]

Roger Muñoz-Navarro[3]

Paloma Ruíz-Rodríguez[4]

Leonardo Adrián Medrano[5]

Antonio Cano-Vindel[6]

[1]Mental Health Centre, University Hospital "Marqués de Valdecilla"- IDIVAL. Santander, Spain.

[2]Faculty of Health Sciences, Universidad Europea del Atlántico, Santander, Spain

[3]Department of Basic Psychology, Faculty of Psychology, University of Valencia, Valencia, Spain

[4]Castilla La Nueva Primary Care Centre, Health Service of Madrid, Madrid, Spain

[5]Faculty of Psychology, University Siglo 21, Córdoba, Argentina

[6]Department of Basic Psychology, Faculty of Psychology, University Complutense of Madrid, Madrid, Spain

Table S1. Correlation matrix of symptoms intensity and QoL domains

QoL (WHOQOL-Bref)	Depression (PHQ-9)	Anxiety (GAD-7)	Somatizations (PHQ-15)
Psychological	-.600[a]	-.477[a]	-.558[a]
Physical	-.668[a]	-.510[a]	-.419[a]
Social relations	-.400[a]	-.241[a]	-.201[a]
Environmental	-.384[a]	-.336[a]	-.302[a]
Overall	-.420[a]	-.301[a]	-.267[a]

[a] Correlation is significant at the 0.01 level.

QoL, Quality of Life; WHOQOL-Bref , World Health Organization Quality of Life Instrument-Short Form; PHQ-9, Patient Health Questionnaire-9; GAD-7, Generalized Anxiety Disorder 7; PHQ-15, Patient Health Questionnaire-15.

Table S2. Covariate Adjusted Means (standard errors) of QoL as a function of the number of mental disorder diagnoses[b]

	**Number of diagnoses**			
	**0** (n= 195)	**1** (n= 261)	**2** (n= 299)	**3** (n= 486)
	Mean (SE)[b]	Mean (SE)	Mean (SE)	Mean (SE)
**QoL (WHOQOL-Bref)[c]**				
Psychological (0-100)	70.65 (0.99)	59.88 (0.85)**	54.44 (0.79)**	44.61 (0.63)**
Physical (0-100)	58.57 (1.05)	52.76 (0.90)**	45.18 (0.84)**	34.93 (0.67)**
Social relations (0-100)	60.47 (1.44)	54.53 (1.23)*	50.09 (1.15)	43.49 (0.91)**
Environmental (0-100)	63.88 (0.98)	59.74 (0.84)*	55.91 (0.78)*	51.27 (0.62)**
Overall (0-4)	3.34 (0.06)	3.10 (0.05)*	2.89 (0.05)*	2.58 (0.04)**

*p<.01; **p<.001
WHOQOL-Bref , World Health Organization Quality of Life Instrument-Short Form; QoL, Quality of Life
[a] Means after controlling for covariates (age, sex, education, marital status, employment status, and income).
[b] Significance in the column ‘1 diagnosis’ refer to the difference with ‘no diagnosis’, in the ‘2 diagnoses’ column, to the difference between 2 diagnoses and 1 diagnosis, and in the ‘3 diagnoses’ column to the difference between 3 diagnoses and 2 diagnoses. Diagnoses based on algorithms from the PHQ-9, GAD-7 and PHQ-15 for major depression, generalized anxiety and somatoform disorders, respectively.
[b] SE- standard errors based on 1000 bootstrap samples.
[c] in parenthesis overall score range of each subscale.
